# Completeness of Community Structure in Networks

**DOI:** 10.1038/s41598-017-05585-6

**Published:** 2017-07-13

**Authors:** Jia-Rong Xie, Pan Zhang, Hai-Feng Zhang, Bing-Hong Wang

**Affiliations:** 1 Department of Modern Physics, University of Science and Technology of China, Hefei, 230026 China; 2 CAS key laboratory of theoretical physics, Institute of Theoretical Physics, Chinese Academy of Sciences, Beijing, 100190 China; 3 School of Mathematical Science, Anhui University, Hefei, 230601 China

## Abstract

By defining a new measure to community structure, *exclusive modularity*, and based on cavity method of statistical physics, we develop a mathematically principled method to determine the *completeness* of community structure, which represents whether a partition that is either annotated by experts or given by a community-detection algorithm, carries complete information about community structure in the network. Our results demonstrate that the expert partition is surprisingly incomplete in some networks such as the famous political blogs network, indicating that the relation between meta-data and community structure in real-world networks needs to be re-examined. As a byproduct we find that the exclusive modularity, which introduces a null model based on the degree-corrected stochastic block model, is of independent interest. We discuss its applications as principled ways of detecting hidden structures, finding hierarchical structures without removing edges, and obtaining low-dimensional embedding of networks.

## Introduction

Community structure, a partition of nodes into groups in such a way that the number of edges within groups is comparatively larger than the number of edges between groups, has attracted great attention over the past decade^1–3^. Many real-world networks, such as social networks^4^, biochemical networks^5^ and information networks^6^, have been shown to possess community structure. Community structure provides a large-scale connecting pattern of networks, hence is very helpful in understanding the macroscopic structure of networks.

Many methods have been proposed to detect the community structure. These include modularity-based methods^7–10^, spectral clustering^11–13^ and statistical inference^14, 15^. For evaluating their performance, those methods are usually validated and compared on two kinds of networks, synthetic benchmark networks with plant-in structure^16–18^, and real-world networks for which there is an expert partition^17, 19, 20^. Despite the wide use of expert partitions in evaluating community detection algorithms, the evaluation of the expert partitions has attracted few attention. Often, the expert partition is considered to describe the most important information regarding the network connectivity and is given by domain experts or based on additional information of the network. However it may be incomplete in describing communities in the network, due to artificial operations to the labels, or some unknown information to the community structures. Then a question arises naturally: how to determine whether the expert partition contains enough information about the structure?

In this paper, we address this problem by studying whether a network contains statistical significant communities when the expert partition is *excluded* from the candidate set of partitions for representing the community structure. Our approach relies on two important ingredients: (1) how to exclude a given partition in detecting communities, and (2) how to determine whether there are structure after the exclusion. In the first we define a new measure to the community structure, namely *Exclusive Modularity* (EM), by using a null model based on the degree-corrected stochastic block model where a given partition is planted; in the second we map the network to a (Potts) spin glass system at finite temperature, use exclusive modularity as negative Hamiltonian and apply statistical mechanics to determine whether system has a retrieval state which represents a statistically significant community structure.

Our ideas results to a message passing algorithm, which has computational complexity linear in system size in sparse networks, thus is scalable to sparse networks with millions of nodes. We apply our method to both synthetic networks and real-world networks such as the karate club network and political blogs network. On the synthetic networks generated by the stochastic block model and on the karate club network, by excluding the planted partition or expert partition, we find that the remaining network has no retrieval state, which indicates that the partitions contain complete information of community structure. While on some other real-world networks such as political blogs network, our algorithm reports that the expert partition is incomplete, showing that there are other information hidden by the expert partition.

Our method can be used to detect such hidden partitions and further the hierarchies which are common in real-world networks. A usually adopted top-bottom way of finding the hierarchies is to iteratively detect sub-communities in the subgraphs composed of nodes in the same communities that already found. This method needs to remove edges connecting different subgraphs, hence certainly drops some information about the structure. We show that by applying our method recursively in a combination-exclusion way, we can give a principled method of finding hierarchies without removing edges connecting different communities.

## Results

### Completeness of the planted partition in the stochastic block model

Our main assumption is that if the given partition {*t*} contains complete information of the community structure in the graph, there should be no other community structures that are detectable after {*t*} is excluded. The existence of such community structure is characterized by the existence of the *retrieval state* as we define in the Method section. A straightforward example to illustrate this is the stochastic block model (SBM), which is a popular ensemble of networks with community structure. In SBM there is a partition {*t*} with *q* groups planted as a ground-true community structure, and edges are generated independently according to a *q* × *q* matrix **P**. Usually we consider the case where **P** has two distinct entries, *P*_*in*_ on the diagonal and *P*_*out*_ on the off-diagonal. Recently, a detectability transition^14^ has been discovered below which no algorithm can detect finite information of the planted partition. It has been shown in ref. 10 that belied propagation algorithm using the classic modularity as negative Hamiltonian works all the way down to the detectability transition of the SBM, as the retrieval phase representing the planted partition in the phase diagram always exists in the detectable phase. Thus as a sanity check to our assumption, excluding the planted partition should remove the retrieval phase completely from the phase diagram, reflecting that there is no other statistically significant structures in SBM except the excluded one.

**Figure 1:**
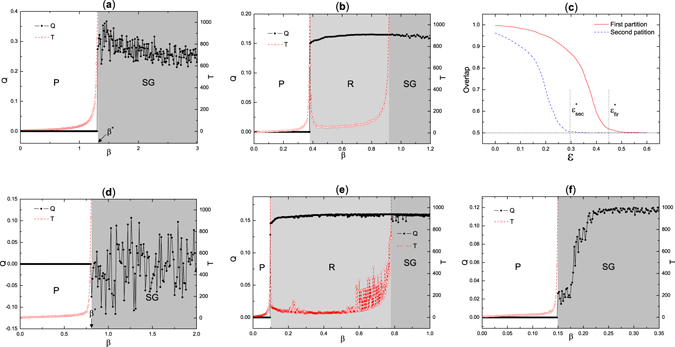
Results of BP in some networks. Exclusive modularity and convergence time given by BP of: (**a**) a network generated by stochastic block model with *N* = 2000 nodes, average degree *c* = 3, *q* = 2 groups and $\varepsilon ={P}_{out}/{P}_{in}=0.1$; (**b**) a network generated by biSBM with *N* = 2000, *c* = 20, *ε* = 0.2 and *α* = 0.7; (**d**) karate club with expert partition excluded; (**e**) political blogs with expert partition excluded; (**f**) political blogs with 6 subgroups excluded. In these figures *P*, *R*, and *SG* denote paramagnetic, retrieval and spin glass phase respectively. (**c**) The overlap (fraction of correctly reconstructed labels) of the two planted partitions given by BP are plotted. Here *c* = 20 and *α *= 0.7. We average over 100 instances for network generated by biSBM with size *N* = 1000. The detectability thresholds ${\varepsilon }_{fir}^{\ast }$ and ${\varepsilon }_{sec}^{\ast }$ are theoretical results.

In Fig. 1(a), on a network generated by the SBM, we plot the exclusive modularity (with the planted partition excluded) and convergence time of *belief propagation* (BP) (see Eqs (4) and (5)). We can see from the figure that there are just two phases, paramagnetic phase defined by 0 exclusive modularity and spin-glass phase defined by in-convergence of BP, separated by a transition at ${\beta }^{\ast }=log[q/(\sqrt{\tilde{c}}-1)+1]$ (see in Method section and Supplementary Information for details on computing this transition), where $\tilde{c}$ is the average excess degree. Clearly there is no retrieval phase exists in the phase diagram, verifying our analysis that the planted partition is complete in this synthetic network.

To illustrate further the effects of partition-exclusion, we propose the biSBM, stochastic block model with two planted partitions (detailed in Supplementary Information), and investigate whether our method can detect the second partition with the first partition being excluded. Analogous to the standard SBM, the biSBM use parameter $\varepsilon ={p}_{out}/{p}_{in}$ to adjust the relative strength of the community structure. In addition to *ε*, biSBM introduces another parameter *α* to control the ratio of edges that belong to two community structures. Obviously, single partition is incomplete in describing the whole community structure in the network, and excluding either partition would leave a retrieval phase representing the other community structure in the phase diagram.

On a network generated by the biSBM, the exclusive modularity (with the first partition excluded) and convergence time are plotted as a function of *β* in Fig. 1(b). We can see from the figure that in between the paramagnetic and spin-glass phases, there is a retrieval phase, where BP converges to a fixed-point with large exclusive modularity, which is correlated with second partition. Thanks to the locally structure of the graph, we claim that our algorithm for finding the second partition is optimal, both in accuracy and in ability of detecting the second partition with a success better than random guess all the way down to the detectability transition (see SI). In Fig. 1(c), the accuracy of detecting the second partition (in overlap, fraction of correctly reconstructed labels) is plotted against parameter *ε*, we can see that BP works all the way down to the detectability transition of both two partitions. In other words, our results indicate that our algorithm does not through away any information of the second partition when excluding the first one.

### Completeness of expert partition in real-world networks

For many real-world networks, for example the famous karate club network^19^ and political blogs network^20^, annotations (i.e. expert partitions) are believed to reflect the true community structure of the network, hence have been widely used in validating community detection algorithms. However few attention has been paid on the validity of the expert partitions. In this section we adopt our method to investigate the completeness of the expert partitions in representing community structure in real-world networks.

Our results are shown in Fig. 1(d)–(f). In the karate club network, we find that the retrieval phase is completely absent when the expert partition is excluded, which indicates that expert partition is complete. While in political blogs Fig. 1(e) we find that after excluding the expert partition, the retrieval phase still presents. It means that the expert partition of separating liberals and conservatives is incomplete in describing the underlying community structure. We note that there are already studies on this network^10, 21^, reporting that in addition to the liberals-conservatives division, there are more groups forming a hierarchical structure with a 4-layer dendrogram. To check the completeness of these results, rather than using the expert partition, we set 6 groups in the top-2 levels of the dendrogram as found in ref. 10 as the excluded partition. Figure 1(f) shows that the retrieval phase disappears, hence there is not hidden community structure. It indicates that the partition with 6 groups contains complete information of the underlying community structure. It has been reported^22^ that much more than 6 groups can be found in the political blogs network using other techniques. The reason that we found 6 groups are enough may be because our current approach only focuses on assortative structures using a positive modularity. A simple extension of our approach to study the dis-assortative structures is running BP with a negative *β*.

Another network we examine is a network of school students drawn from the US National Longitudinal Study of Adolescent to Adult Health, which consists of students of a high school (US grades 9 to 12) and its feeder middle school (grades 7 and 8). In the dataset, the network structure is accompanied by the annotations of gender, ethnicity and school grade. We find that the retrieval phase presents after the partition given by the annotations (which is combined by three properties) is excluded. It means that the information of annotations are not enough for characterizing community structure of the network.

### Other applications of the exclusive modularity

It is straightforward to see our method not only determines the completeness of a partition {*t*}, but also gives a new partition {*g*} as a complement to {*t*}, which is the marginalized partition in the retrieval state: ${g}_{i}={\rm{\arg }}\,{{\rm{\max }}}_{h}{\psi }_{h}^{i}$. We call this process *detecting hidden partitions*, with “hidden” reflecting the fact that {*g*} is essentially covered by {*t*} and can be detected only when {*t*} is excluded. Actually we notice that any community detection method (explicitly or implicitly) excludes the partition which puts all nodes into one group, as this “ferromagnetic partition” is a strong yet valid solution to the community detection task, but is obviously unwanted.

**Figure 2:**
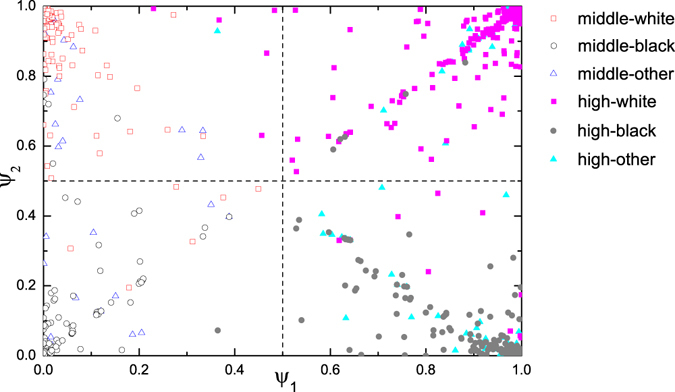
The marginals of BP in the network of school students before and after the first partition is excluded. The X-axis (Y-axis) is the marginal of nodes in first (second) partition with 2 groups. The real classification of high/middle school are indicated by solid/hollow nodes. While ethnicity of white/black/other are indicated by square/circular/triangular nodes. And they are also indicated by different colors. The vertical and horizontal dash lines are the boundaries of the two detected partitions.

This process of excluding can be naturally used as an embedding of a network into a low-dimensional space using marginals of BP. We again take the network of school students as an example. In the X-axis of Fig. 2 we plot the marginal probability of node *i* being in the the first group ${{\rm{\Psi }}}_{1}^{i}$ (we use *q* = 2 groups, so simply the probability of it being in another group is $1-{{\rm{\Psi }}}_{1}^{i}$) with no partition excluded (i.e. using classic modularity). In Y-axis we plot the marginal probability ${{\rm{\Psi }}}_{2}^{i}$ given by BP with the first detected partition being excluded. From the figure one can see that the first detected partition can recover the expert partition (the classification of high/middle school) very precisely: almost all students in middle (high) school are placed at the left (right) side of the vertical boundary (the vertical dash line at Ψ = 0.5). And the second partition, represented by Ψ_2_, can recover the ethnicity very well.

Low-dimensional embedding, using e.g. spectral methods, is very helpful in understanding and visualizing large networks^23^. However to our best knowledge our method is first one that uses marginals of a message-passing algorithm for embedding. Our method is superior to existing methods in the sense that BP marginals gives close-to-optimal results in detection accuracy in stochastic block model, while existing ones such as spectral methods usually can be seen as a linear approximation to the optimal message passing ones^13^, hence are sub-optimal.

The partition we want to exclude, {*t*}, is not necessarily assortative (as in the planted partition or expert division), but could be of any kind. Once we have found partition {*g*} by excluding {*t*}, we can combine {*g*} and {*t*} to a partition {*h*} = {*g*}⊗{*t*}^24^, then find a new partition by running BP algorithm excluding {*h*}. By doing this combining-excluding procedure iteratively we are able to find hierarchical structures layer by layer in the dendrogram. There are many algorithms exiting for finding hierarchical structure in a top-down way. The standard procedure is building subgraphs at each level of dendrogram, using nodes in the same communities of the upper level in the dendrogram by removing edges between subgraphs, then running community detection on the subgraphs. This standard process is slow as community algorithm has to be ran in each subgraph. Moreover removing edges certainly drops some information about the community structures. Our method is obviously more efficient than existing top-down methods in finding hierarchies, because each run of BP finds one layer in the dendrogram; and more accurate, because our method do not remove any edges.

## Discussion

We have presented a method for validating completeness of a given partition in characterizing community structure in networks. We defined the exclusive modularity by excluding the given partition, and proposed an efficient belief propagation algorithm using the exclusive modularity as Hamiltonian to determine whether there is a retrieval phase in the system representing statistically significant community structure, that implies the incompleteness of the community structure.

We applied our method to validate expert partitions in real-world networks. Our results reveal that in some networks, such as the karate club network, the expert partition is complete in describing the community structure, while in some other networks, such as political blogs network, the expert division is incomplete, indicating that there are some hidden structures that are ignored by the expert division. We believe our method gives a principled way to examine the relation between meta-data of networks and large-scale structures in networks. In addition to the completeness validation, we also discussed applications of our method in detecting hidden structures, finding hierarchical structures without removing edges, and obtaining low-dimensional embedding of networks.

There have been many work discussing the statistically significance of community structures. These include examining the likelihood ration^25^, using the Bethe free energy^14^ and adopting the minimum description length^21^. The difference between our method and the others is that it combines both the classic measure of community structure, the modularity, and Bayesian statistics. In essence, our algorithm looks for consensus of many good partitions, rather than a single best one.

A possible extension of our method to finding structures more than assortative ones, such as core-periphery structures, is to adopt the inference of the stochastic block model with a given partition excluded, i.e. treated as a null model. We will put this into future work.

## Method

The core idea of this work is to study the statistical mechanics of community detection by excluding a partition, {*t*}. We do this by giving each partition {*g*} an exclusive modularity *Q*({*g*}|{*t*}), as a measure of community structure

1 $$Q(\{g\}|\{t\})=\frac{1}{2M}\sum _{i,j}\,[{A}_{ij}-{p}_{ij}(\{t\})]{\delta }_{{g}_{i}{g}_{j}},$$

where *p*_*ij*_({*t*}) is expected probability of node *i* connecting node *j* in a model described as follows: suppose *i* and *j* belong to group *r* and *s* of partition {*t*} with *t*_*i*_ = *r* and *t*_*j*_ = *s* respectively. Let ${p}_{i|sr}$ be the probability that node *i* is the endpoint of one edge randomly chosen from the edges connecting groups *r* and *s*. Therefore one has ${p}_{i|sr}={k}_{is}/{M}_{rs}$, where ${k}_{is}={\sum }_{j}{A}_{ij}{\delta }_{{t}_{j}s}$ is the number neighbors of node *i* in group *s*, and *M*_*rs*_ is the total number of edges connecting groups *r* and *s*, which satisfies ${M}_{rs}={\sum }_{i}{k}_{is}{\delta }_{{t}_{i}r}$ (if *r* = *s*, *M*_*rr*_ is the twice number of edges within group *r* because each edge is counted twice in the sum). Similarly, we have ${p}_{j|rs}={k}_{jr}/{M}_{rs}$. By making a first-order approximation one has:

2 $${p}_{ij}(\{t\})\sim {p}_{i|sr}{p}_{j|rs}{M}_{rs}=\frac{{k}_{i{t}_{j}}{k}_{j{t}_{i}}}{{M}_{{t}_{i}{t}_{j}}}$$

It is easy to see that the probability *p*_*ij*_({*t*}) is proposed as if it is the planted partition of (a variant of) the degree-corrected stochastic block model (DCSBM)^15^. In this sense, the exclusive modularity uses a DCSBM (with a given partition being the planted partition) as a null model, as opposed to the classic modularity^7^ which uses a configuration model as a null model. As a consequence, the classic modularity can be seen as a special case of exclusive modularity that excludes the all-one vector. It is straightforward to see that *Q*({*t*}|{*t*}) = 0, which gives a simple check that the partition {*t*} is indeed excluded from our consideration of community structure. For a partition {*g*}, a larger exclusive modularity *Q*({*t*}) reveals that {*g*} gives more internal edges than the the expected number of internal edges in random partitions (except the excluded one {*t*}), hence is more likely to represent the underlying community structure.

As pointed out by ref. 10, directly maximizing the modularity is prone to overfitting, finding partitions with high modularity even in random networks. In this sense, simply maximizing an objective function can not answer the model selection problem on whether the hidden community structure exists. Thus, we need to add some notion of statistical significance. Here we generalize the method proposed in ref. 10 which uses belief propagation algorithm to find consensus of many high modularity partitions, to our case that uses exclusive modularity as a measure of community structure. In more detail, we tackle the problem of determining whether there exist statistically significant communities using ideas from spin glass theory of statistical physics. We assign to each partition a Hamiltonian equal to negative exclusive modularity, then give a Gibbs-Boltzmann distribution to each partition at a finite temperature

3 $$P(\{g\}|\{t\})=\frac{1}{Z}{e}^{\beta MQ(\{g\}|\{t\})},$$

where *Z* is the partition function and *β* denotes the inverse temperature. Then we scan whole range of *β* to look for a phase which indicates the existence of a significant community structure with partition {*t*} excluded.

Although above Boltzmann distribution is difficult to solve in general, on the sparse graphs we can efficiently solve the marginals approximately using BP, which computes marginals probability of node *i* being in group *h* as

4 $${\psi }_{h}^{i}=\frac{1}{{Z}_{i}}{e}^{-\beta \sum _{s}{k}_{is}{\chi }_{h|{t}_{i}s}}\prod _{k\in \partial i}[1+({e}^{\beta }-1){\psi }_{h}^{k\to i}],$$

where *Z*_*i*_ is the normalization factor, *t*_*i*_, *r*, *s* are the groups of {*t*}, *h* is the group of detected partition {*g*}, ${\chi }_{h|rs}={\sum }_{l}{k}_{lr}{\psi }_{h}^{l}{\delta }_{{t}_{l}s}/{M}_{rs}$ denotes the external field acting on nodes in group *h*, and ${\psi }_{h}^{k\to i}$ denotes the (cavity) probability of node *k* being in group *h* when node *i* is removed from the graph. The cavity probabilities (so-called BP messages) along directed edges of the graph can be determined by BP iterating equation

5 $${\psi }_{h}^{i\to j}=\frac{1}{{Z}_{i\to j}}{e}^{-\beta \sum _{s}{k}_{is}{\chi }_{h|{t}_{i}s}}\prod _{k\in \partial i\backslash j}[1+({e}^{\beta }-1){\psi }_{h}^{k\to i}]\mathrm{.}$$

Here *Z*_*i→j*_ is the normalization factor, ∂*i*\*j* is the set of neighbors of *i* except *j*. On a network, we iterate BP equations Eq. (5) till converge, or stop after a given maximum number of iterations is exceeded, then compute marginals using Eq. (4), marginalized partition $\{\hat{t}\}$ and its exclusive modularity. Using the convergence time and exclusive modularity of partition $\{\hat{t}\}$, we can further separate system into different phases as studied in ref. 10.

As we have emphasized, we are mostly interested in whether there exists a significant structure with a large exclusive modularity, at a certain temperature. This is amount to finding an extremal Gibbs state at a certain temperature where the spin-glass susceptibility does not diverge. On sparse networks the divergence of susceptibility is characterized by in-convergence of BP. So in practice we just need to look for a *retrieval state* where BP converges and BP messages non-trivial. A simple way to do this is by scanning whole range of *β*, or using a binary search. Actually as pointed out in ref. 10, one only needs to check the convergence property and retrieval modularity at a critical temperature ${\beta }^{\ast }=log[q/(\sqrt{\tilde{c}}-\mathrm{1)}+1]$. When *β* < *β* ^*^, the paramagnetic fixed point ${\psi }_{h}^{i\to j}={\psi }_{h}^{i}=\frac{1}{q}$ is locally stable, hence system is in the paramagnetic phase. With *β* > *β* ^*^, the paramagnetic fixed point becomes unstable under random perturbations, and system either enters the spin glass phase, or retrieval phase if it exists. Thus the spin glass transition *β* ^*^ can be estimated by studying local instability of the factorized fixed point in the under the locally-tree-like assumptions, as detailed in Sec.V of Supplementary Information.

## Electronic supplementary material

Supplementary Information
