# Supplementary material for: Completeness of Community Structure in Networks
Source: Sci Rep. 2017 Jul 13;7:5269. doi: 10.1038/s41598-017-05585-6 (PMC5509661; doi:10.1038/s41598-017-05585-6)
Supplement: Supplementary file 1 — Supplementary Information [file 41598_2017_5585_MOESM1_ESM.pdf]

Supplementary Information: Completeness of Community Structure in Networks

Jia-Rong Xie, Pan Zhang, Hai-Feng Zhang, and Bing-Hong Wang

I. COMPARISON BETWEEN DCSBM AND ITS VARIANT

In the main text, we propose a variant of degree-corrected stochastic block model (DCSBM):

$$p_{ij}\left(\{t\}\right) = \frac{k_{it_j} k_{jt_i}}{M_{t_i t_j}}. \tag{S1}$$

A substitution is DCSBM [1]:

$$\widetilde{p}_{ij}\left(\{t\}\right) = k_i k_j \frac{M_{t_i t_j}}{\kappa_{t_i} \kappa_{t_j}}, \tag{S2}$$

where $\kappa_r = \sum_i k_i \delta_{t_i r}$ is sum of degree of nodes in group r. DCSBM satisfies the constraints:

$$\begin{cases} \sum\limits_j \widetilde{p}_{ij}\left(\{t\}\right) = k_i \\ \sum\limits_{ij} \widetilde{p}_{ij}\left(\{t\}\right) \delta_{t_i r} \delta_{t_j s} = M_{rs} \end{cases}. \tag{S3}$$

While the variant satisfies:

$$\sum_j p_{ij}\left(\{t\}\right) \delta_{t_j s} = k_{is}, \tag{S4}$$

which include the constraints in Eq. (S3). Therefore, the variant is more stricter and more accurate.

II. THE EXCLUSIVE MODULARITY OF A PARTITION EXCLUDING ITSELF

The exclusive modularity can be written in another form:

$$\begin{aligned} Q\left(\{g\}|\{t\}\right) &= \sum_h \frac{M_{hh}}{2M} - \sum_h \sum_{r,s} \sum_{i,j} \frac{k_{is} k_{jr}}{M_{rs}} \delta_{t_i r} \delta_{g_i h} \delta_{t_j s} \delta_{g_j h} \\ &= \sum_h \frac{M_{hh}}{2M} - \sum_h \sum_{r,s} \frac{M_{rh,s} M_{sh,r}}{2M M_{rs}}. \end{aligned} \tag{S5}$$

Here, t_i,r, s denote the groups of partition $\{t\}$ and g_i, h, denote groups of partition $\{g\}$. $M_{rh,s} = \sum\limits_i k_{is} \delta_{t_i r} \delta_{g_i h} = \sum\limits_{i,j} A_{ij} \delta_{t_i r} \delta_{g_i h} \delta_{t_j s}$ is the number of edges connecting two subsets of nodes, one subset is the intersection of groups r and h, the other subset is group s. But the edges whose both sides are in the intersection of the two subsets should be counted twice.

If the two partitions are the same, then we have $M_{rh,s} = M_{rs}\delta_{rh}$ and $M_{sh,r} = M_{rs}\delta_{sh}$, Eq. (S5) leads to:

$$Q\left(\{t\}|\{t\}\right) = \sum_h \frac{M_{hh}}{2M} - \sum_h \sum_{r,s} \frac{M_{rs} \delta_{rh} \delta_{sh}}{2M} = 0, \tag{S6}$$

which gives a simple check that the partition $\{t\}$ is indeed excluded from our consideration of community structure.

III. DESCRIPTIONS OF BISBM

Stochastic block model (SBM) [2] is a model of synthetic networks with one planted partition. We here just introduce the simplest SBM, in which nodes are randomly assigned to two groups. For each node i, the label $t_i \in \{1, 2\}$ indicates its assignment. The probability of node i connecting to j depends only on the labels they have, i.e., $p_{ij} = P_{t_i t_j}$. In the simplest case, $P_{11} = P_{22} = P_{in} = 2p/(1+\epsilon)$ and $P_{12} = P_{21} = P_{out} = 2p\epsilon/(1+\epsilon)$. Define $\epsilon = P_{out}/P_{in}$ be the strength of tendency that nodes prefer to connect to the nodes with the same label to them. Thus, smaller value of ϵ gives rise to stronger community structure. $p = c/N$ denotes the strength of connection and c is the average degree. The probability p_{ij} can be written in a uniform way:

$$p_{ij} = p + \frac{1-\epsilon}{1+\epsilon}(2\delta_{t_i t_j} - 1)p. \tag{S7}$$

The SBM is the generative model with one partition. Here, we propose a special case, biSBM. We just show the simplest case: each node has two labels correspond to the two partitions, $t_i \in \{1, 2\}$ (first partition) and $g_i \in \{①, ②\}$ (second partition). Let p^{fir} (p^{sec}) be the strength of connection of the first (second) partition, and $\alpha = p^{sec}/p^{fir}$ be the bias attachment strength between the two partitions. Then the probability of p_{ij} in biSBM is given as:

$$\begin{aligned} p_{ij} &= p^{fir} + \frac{1-\epsilon^{fir}}{1+\epsilon^{fir}}(2\delta_{t_i t_j} - 1)p^{fir} + p^{sec} + \frac{1-\epsilon^{sec}}{1+\epsilon^{sec}}(2\delta_{g_i g_j} - 1)p^{sec} \\ &= p + \frac{1}{1+\alpha}\frac{1-\epsilon^{fir}}{1+\epsilon^{fir}}(2\delta_{t_i t_j} - 1)p + \frac{\alpha}{1+\alpha}\frac{1-\epsilon^{sec}}{1+\epsilon^{sec}}(2\delta_{g_i g_j} - 1)p, \end{aligned} \tag{S8}$$

where $p = p^{fir} + p^{sec}$. ϵ^{fir} (ϵ^{sec}) is the strength of tendency of first partition (second partition). By assuming $\epsilon^{fir} = \epsilon^{sec} = \epsilon$, Eq. (S8) can be rewritten as:

$$p_{ij} = p + \frac{1}{1+\alpha}\frac{1-\epsilon}{1+\epsilon}(2\delta_{t_i t_j} - 1)p + \frac{\alpha}{1+\alpha}\frac{1-\epsilon}{1+\epsilon}(2\delta_{g_i g_j} - 1)p. \tag{S9}$$

We only consider the case $0 \le \alpha \le 1$, $0 \le \epsilon \le 1$ and $c > 1$.

IV. DETECTABILITY THRESHOLD OF BISBM

The phase diagram of SBM can be calculated by spectral methods [3, 4] to determine the detectability threshold of planted partition. Here we generalize the method to biSBM to obtain the detectability thresholds of the first partition and the second partition. The derivations are exact only if c is very large.

Firstly, we calculate the spectrum of ensemble of adjacency matrix $\mathbf{A}$ of networks generated by biSBM. Since $\mathbf{A}$ is symmetric, the eigenvalues are all real. It can be written in the form

$$\mathbf{A} = \mathbf{X} + \mathbf{P} = \mathbf{X} + c\mathbf{1}\mathbf{1}^T + c\frac{1}{1+\alpha}\frac{1-\epsilon}{1+\epsilon}\mathbf{u}\mathbf{u}^T + c\frac{\alpha}{1+\alpha}\frac{1-\epsilon}{1+\epsilon}\mathbf{v}\mathbf{v}^T, \tag{S10}$$

where $\mathbf{1}$, $\mathbf{u}$ and $\mathbf{v}$ are unit vectors $\mathbf{1} = (\underbrace{1, 1, ..., 1}_{N})^T/\sqrt{N}$, $\mathbf{u} = (\underbrace{1, 1, ..., 1}_{N/2}, \underbrace{-1, -1, ..., -1}_{N/2})^T/\sqrt{N}$ and $\mathbf{v} = (\underbrace{1, 1, ..., 1}_{N/4}, \underbrace{-1, -1, ..., -1}_{N/4}, \underbrace{1, 1, ..., 1}_{N/4}, \underbrace{-1, -1, ..., -1}_{N/4})^T/\sqrt{N}$, the elements of $\mathbf{P}$ are p_{ij}. The first term $\mathbf{X}$ is a random matrix reflect the deviation between the adjacency matrix and its average value. The spectrum of $\mathbf{X}$ satisfies the Wigner's semicircle law [5] with radius $2\sqrt{c}$:

$$\rho(\lambda) = \frac{\sqrt{4c - \lambda^2}}{2\pi c}. \tag{S11}$$

The second term $c\mathbf{1}\mathbf{1}^T$ reflects the average degree. The first two terms constitute the Erdős-Rényi (ER) model. With this additional term, every eigenvalues increase but not higher than the next one of matrix $\mathbf{X}$. The eigenvalues of $\mathbf{X}$ are tight, so they are almost unchanged. The only exception is the highest one increases to $\lambda_1 = c + 1$, which is separate from the rest of the spectrum. The third term reflects the first partition. The first three terms constitute

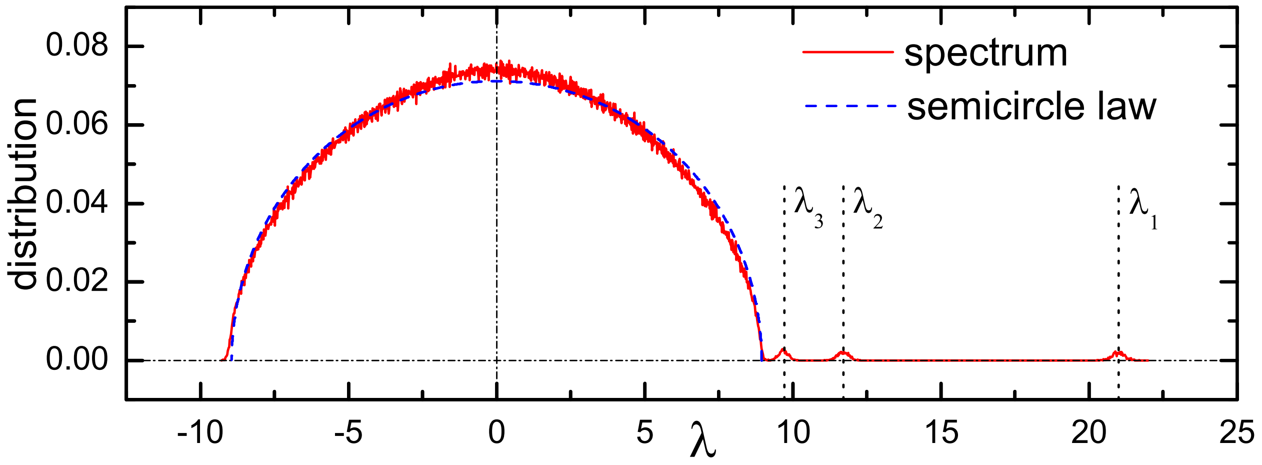

FIG. S1: **Spectrum of adjacency matrices for ensemble of networks generated by biSBM.** Here $c = 20$, $\alpha = 0.7$ and $\epsilon = 0.1$. The distribution is a statistical result of 1000 instances with network size $N = 1000$. The semicircle law and the eigenvalues λ_1, λ_2, λ_3 are theoretical results.

SBM. Similarly, with this additional term, the spectrum is almost unchanged, except for the second eigenvalue, $\lambda_2 = \frac{1-\epsilon}{(1+\alpha)(1+\epsilon)}c + \frac{(1+\alpha)(1+\epsilon)}{1-\epsilon}$ (it presents only if $\frac{1-\epsilon}{(1+\alpha)(1+\epsilon)} > \frac{1}{\sqrt{c}}$). The eigenvector of λ_2 corresponds to the first partition. All results above have been calculated in Ref. [3]. The only difference of adjacency matrices between SBM and biSBM is the addition of the forth term, which reflects the second partition. Similarly, $\lambda_3 = \frac{\alpha(1-\epsilon)}{(1+\alpha)(1+\epsilon)}c + \frac{(1+\alpha)(1+\epsilon)}{\alpha(1-\epsilon)}$, which presents only if $\frac{\alpha(1-\epsilon)}{(1+\alpha)(1+\epsilon)} > \frac{1}{\sqrt{c}}$. The eigenvector of λ_3 corresponds to the second partition. Fig.S1 shows the spectrum of adjacency matrices for ensemble of biSBM.

If c and α are fixed, the detectability threshold for the first partition or the second partition can be calculated by the condition whether λ_2 or λ_3 to be present, respectively. Thus,

$$\begin{cases} \epsilon^*_{fir} = \dfrac{\sqrt{c}-1-\alpha}{\sqrt{c}+1+\alpha} \\ \epsilon^*_{sec} = \dfrac{\alpha\sqrt{c}-1-\alpha}{\alpha\sqrt{c}+1+\alpha} \end{cases}. \tag{S12}$$

And the bulk of spectrum of nonbacktracking matrix $\mathbf{B}$ [4] for biSBM is confined to the disk in the complex plane with radius $\sqrt{c}$. And at most three real eigenvalues outside the bulk, $\lambda_1^{\mathbf{B}} = c$, $\lambda_2^{\mathbf{B}} = \frac{1-\epsilon}{(1+\alpha)(1+\epsilon)}c$ and $\lambda_3^{\mathbf{B}} = \frac{\alpha(1-\epsilon)}{(1+\alpha)(1+\epsilon)}c$. The conditions that they present are the same to the adjacency matrix $\mathbf{A}$. Fig. S2 shows the spectrum of nonbacktracking matrix for a network generated by biSBM.

The details of the calculation of spectrum of SBM are shown in [3, 4]. The derivation of biSBM is similar and is not shown here.

V. DETAILS OF THE PHASES

In this paper, a partition is analogous to a microstate in statistical physics. At different temperature, the system will be in one of the three macrostates: paramagnetic, retrieval or spin-glass state.

Observe that the factorized solution $\psi_h^i = 1/q$ and $\psi_h^{i\to j} = 1/q$ is always a fixed point of BP (see Eq. (4) and (5) in the main text). If BP converges to this solution, we cannot label the nodes better than chance, and the exclusive modularity is zero. This is the paramagnetic state. At high temperature, the probability of each partition is almost equal, the system is in the paramagnetic state.

BP may converge to a nonfactorized fixed point, which is called the retrieval state [6]. In retrieval state, BP finds the partition corresponding to hidden community structure. The system may in the retrieval state at finial temperature. In this paper, we mainly determine whether the retrieval phase exists.

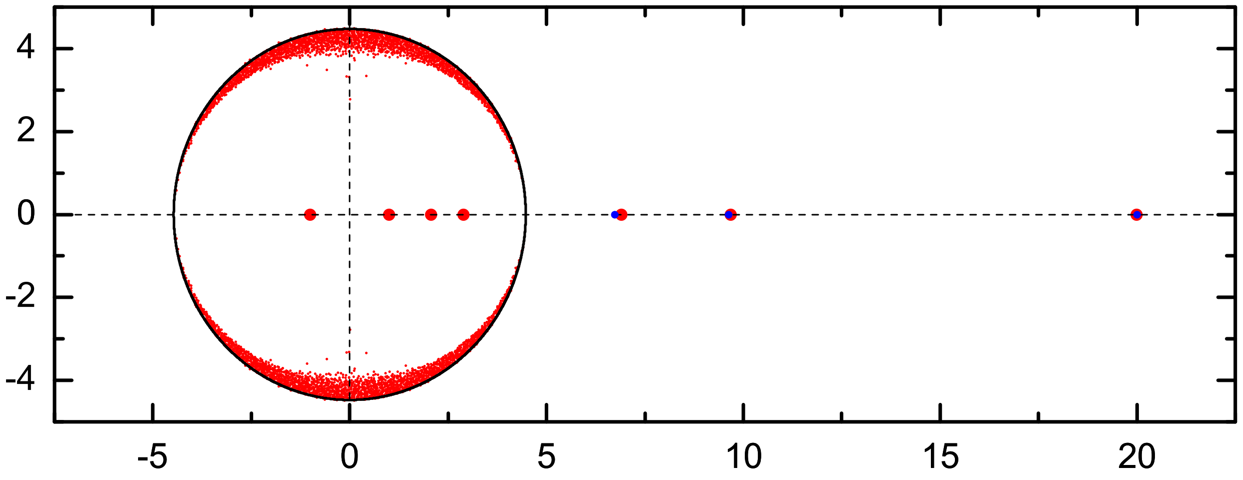

FIG. S2: **Spectrum of nonbacktracking matrix in the complex plane for a network generated by biSBM.** Here $c = 20$, $\alpha = 0.7$, $\epsilon = 0.1$ and $N = 2000$. The red dots are the eigenvalues. The dots with large size are real eigenvalues. The blue dots are theoretical eigenvalues $\lambda_1^{\mathbf{B}}$, $\lambda_2^{\mathbf{B}}$ and $\lambda_3^{\mathbf{B}}$ from right to left. The radius of the circle is $\sqrt{c}$.

At low temperature, BP fails to converge, the system is in the spin-glass state. In this state, neither the factorized solution nor any other fixed point is locally stable.

Therefore, the transition temperature between the paramagnetic phase and the spin-glass phase can be analyzed by the stability of factorized solution. To be specific, by computing the derivatives of messages with respect to each other. At factorized fixed point, we have $\chi_{h|rs} = 1/q$ and $\sum_s k_{is}\chi_{h|t_is} = k_i/q$. We find that $\partial\psi_g^{i\to j}/\partial\psi_h^{k\to i}\mid_{1/q}= T_{gh}$, where T_{gh} is the element of a $q \times q$ matrix

$$T_{gh} = \frac{e^\beta - 1}{e^\beta - 1 + q}\left(\delta_{gh} - \frac{1}{q}\right). \tag{S13}$$

Its largest eigenvalue (in magnitude) is $\lambda = \left(e^\beta - 1\right)/\left(e^\beta - 1 + q\right)$. On locally tree-like graphs with Poisson degree distributions and average degree c, the factorized fixed point is then unstable with respect to random noise whenever $c\lambda^2 > 1$. This is also known as the Almeida-Thouless local stability condition [7]. This leads the transition point to be

$$\beta^* = \log\left(\frac{q}{\sqrt{c} - 1} + 1\right). \tag{S14}$$

If the network has some other degree distribution but is otherwise random, Eq. (S14) holds where c is the average excess degree. But the political blogs network with 6 groups excluded does not satisfy Eq. (S14) well, the reason of which is still unknown.

VI. THE NETWORK OF SCHOOL STUDENTS

The network of school students we study is drawn from the US National Longitudinal Study of Adolescent to Adult Health [8]. The students are asked to list their best 5 male and 5 female friends in the school. We construct the undirected networks in which edges between two students present if one of them regards another as one of best friends. The giant component includes 1127 students. The initial data of ethnicity is a classification of 6 groups, white, black, Hispanic, Asian, mixed/other, and missing. The last four groups are in small numbers, we merge them into one group, the other. The numbers of detected groups are determined manually. The modularity and exclusive modularity of detected partitions are $Q(\{t\}) = 0.464$, $Q(\{t\}|\{g\}) = 0.463$, $Q(\{g\}) = 0.436$ and $Q(\{g\}|\{t\}) = 0.435$.

Such a network is somewhat special, the modularity of the two partitions are so close that the two partitions exchange in some instances.

[1] B. Karrer and M. E. J. Newman, Phys. Rev. E 83, 016107 (2011).
[2] P. W. Holland, K. B. Laskey and S. Leinhardt, Social Networks 5, 109 (1983).
[3] R. R. Nadakuditi and M. E. J. Newman, Phys. Rev. Lett. 108, 188701 (2012).
[4] F. Krzakala, C. Moore, E. Mossel, J. Neeman, A. Sly, L. Zdeborová and P. Zhang, Proc. Natl. Acad. Sci. U. S. A. 110, 20935 (2013).
[5] E. P. Winger, Ann. Math. 67, 325 (1958).
[6] P. Zhang and C. Moore, Proc. Natl. Acad. Sci. 111, 18144 (2014).
[7] J. R. L. de Almeida and D. J. Thouless, J. Phys. A 11, 983 (1978).
[8] Data is obtained from Add Health: http://www.cpc.unc.edu/addhealth.
